# Dr. C. Carleton Smith’s Nuggets

**Published:** 1891-02

**Authors:** S. Lilienthal


### DR. C. CARLETON SMITH'S NUGGETS.

EDITORS HOMŒOPATHIC PHYSICIAN:

In the excellent nuggets of Dr. C. C. Smith, that great teacher says, under Phosphorus: The acute chest pains are generally worse on right side, or by lying on right side. Hering, in *Guiding Symptoms*, VIII, 362, teaches: Stitches in left chest, better lying on left side. Again Smith: Worse by least pressure on intercostal muscles, while Hering has pain in chest when coughing, relieved by external pressure, which agrees with the symptom, "the patient when coughing, holds his abdomen with both hands."

In my third edtion of *Homœopathic Therapeutics*, page 877, I remark, in right sided broncho-pneumonia, Phos. is worse from lying on left side, and I want to ask Dr. Smith to solve this question. I think Smith and Hering right, when we take the pathological condition in view, which causes this aggravation or amelioration, because the patient needs all the air and oxygen he can get to breathe, and will prefer that position favorable to easier breathing.

The *Cyclopædia of Drug Pathogenesy* mentions that Holcombe (a reliable prover), had fast shooting pains in right chest and *fugitive pains in both thorax and abdomen*, and one might say all over the body. Materia medica *pura!!!*

Yours in sorrow,

S. Lilienthal.

